# Optical Properties and Growth Characteristics of 8-Quinolinolato Lithium (Liq) Nano-Layers Deposited by Gas Transport Deposition

**DOI:** 10.3390/mi15091089

**Published:** 2024-08-28

**Authors:** Alexandros Zachariadis, Michalis Chatzidis, Despoina Tselekidou, Olaf Wurzinger, Dietmar Keiper, Peter K. Baumann, Michael Heuken, Kyparisis Papadopoulos, Argiris Laskarakis, Stergios Logothetidis, Maria Gioti

**Affiliations:** 1 Nanotechnology Lab LTFN, Department of Physics, Aristotle University of Thessaloniki, 54124 Thessaloniki, Greece; mchatzid@physics.auth.gr (M.C.); detselek@physics.auth.gr (D.T.); kypapado@physics.auth.gr (K.P.); alask@physics.auth.gr (A.L.); logot@auth.gr (S.L.); 2 AIXTRON SE, 2 Dornkaulstr, Herzogenrath, 52134 Aachen, Germany; o.wurzinger@aixtron.com (O.W.); d.keiper@aixtron.com (D.K.); p.baumann@aixtron.com (P.K.B.); m.heuken@aixtron.com (M.H.); 3 Organic Electronic Technologies (OET), 20 Km Thessaloniki-Tagarades Road, 57001 Thessaloniki, Greece

**Keywords:** 8-Quinolinolato Lithium, Liq, Spectroscopic Ellipsometry, OLED, OVPD

## Abstract

Organometallic complexes containing reactive alkali metals, such as lithium (Li), represent a promising material approach for electron injection layers and electron transport layers (EILs and ETLs) to enhance the performance of Organic Light-Emitting Diodes (OLEDs). 8-Quinolinolato Lithium (Liq) has shown remarkable potential as an EIL and ETL when conveyed in very thin films. Nevertheless, the deposition of nano-layers requires precise control over both thickness and morphology. In this work, we investigate the optical properties and morphological characteristics of Liq thin films deposited via Organic Vapor Phase Deposition (OVPD). Specifically, we present our methodology for analyzing the measured pseudodielectric function <ε(ω)> using Spectroscopic Ellipsometry (SE), alongside the nano-topography of evaporated Liq nano-layers using Atomic Force Microscopy (AFM). This information can contribute to the understanding of the functionality of this material, since ultra-thin Liq interlayers can significantly increase the operational stability of OLED architectures.

## 1. Introduction

Organic Light-Emitting Devices (OLEDs) have emerged as one of the most promising environmentally friendly technologies for future lighting solutions. In contrast to traditional light sources, OLEDs offer several remarkable benefits, including an ultra-thin design, the ability to be fabricated on flexible substrates using wet chemical methods, printing techniques (such as inkjet or roll-to-roll coating), and gas transport processes. Additionally, they are characterized by low energy consumption and uniform light emission [1,2,3,4,5,6].

Efficient OLED devices demand low work function metals as cathodes for effective current injection and therefore low consumption operation. Commonly used materials such as Ca are unstable in the ambient environment due to oxidation, which leads to fast deterioration of the device performance and lifetime [3,7]. On the other hand, environmentally stable metals (e.g., Al and Ag) form large injection barriers with the polymer’s lowest unoccupied molecular orbital (LUMO) level, which hampers the injection of electrons. A very thin interlayer between the electrode and the semiconductor can tune the energy levels, promoting the electron injection on the active layer [6,8,9].

A popular approach of electron injection materials is organometallic complexes with reactive alkali metals such as Li [10,11]. 8-Quinolinolato Lithium (Liq), an organometallic compound with a low deposition temperature, is particularly effective as an electron injection material because of the weak binding energy between lithium and the hydroxyquinoline ligand [9,11,12].

The charge carrier transport layers should accomplish the minimum achievable thickness of a planar layer, as the nanostructure at an electrode/organic interface can significantly impact the operating voltage and the interface stability [13]. The optimum thickness of Liq ETL is reported to be less than 2 nm [6,7,9,10,12,13,14]. Undoubtedly, this requires strict control of the process parameters to regulate both the thickness and morphology of the deposited layers. Additionally, it demands reliable and standardized characterization techniques and methodologies.

In terms of device fabrication, Organic Vapor Phase Deposition (OVPD) is one of the most promising manufacturing methods for the precise and reproducible fabrication of OE devices. Key factors for ensuring consistent large-scale production include the precise regulation of nitrogen carrier gas, maintaining a stable process temperature, and controlling the process pressure. Compared to other deposition technologies (such as vacuum thermal evaporation), OVPD offers essential advantages such as high material yield and uniform deposition on large areas [15,16,17].

Among the techniques that can determine the thickness of thin films with nanometer precision, Spectroscopic Ellipsometry (SE) is widely used due to its non-invasive, non-intrusive, and non-destructive nature. However, accurately determining such low thickness values, especially in layered structures, requires knowledge of a precise set of optical constants.

In this work, we perform a spectroscopic study for the optical properties of Liq thin films grown by the OVPD method on c-Si substrates. We present the methodology for analyzing the measured pseudodielectric function <ε(ω)> obtained by SE of Liq thin films. Through this analysis, the film thickness is calculated. In addition, since the surface morphology of the ETL layer is an important parameter that should be accounted for when introducing interfacial layers into OLED devices, we investigate the growth and structural surface characteristics of OVPD Liq nano-layers. These results can enhance the comprehension of the material’s functionality, as ultra-thin Liq interlayers have the potential to notably enhance the operational stability of OLED architectures.

## 2. Materials and Methods

### 2.1. Materials

Commercially available crystal silicon polished wafers were cut into 4 × 4 cm pieces and used as substrates. The substrates were mounted on top of 200 × 200 mm square glass, which is the standard size of the AIXTRON/APEVA OVPD industrial sample holder. 8-hydroxyquinolinolato-lithium of >99% purity (sublimed) from Jilin was used.

### 2.2. Thin Film Fabrication

The deposition of Liq thin film layers was performed by OVPD at the AIXTRON/APEVA labs. The organic materials were sublimed from specially designed heated source containers and then transported by a nitrogen gas stream to the cooled substrate to enable deposition. The deposition rate could be controlled by regulating the carrier gas flow with standard mass flow controllers. The Liq thin films were deposited on Si wafers (100) placed on industrial size square glass substrates with a size of 200 × 200 mm. The deposition rate was fixed at 1 Å/s. The deposition of the Liq samples was measured during the deposition process with a quartz crystal microbalance method (QCM) based on calibration runs with a standard model ellipsometrical analysis. In total, 5 Liq samples (Table 1) were prepared with thicknesses of 2, 5, 10, 30, and 50 nm.

### 2.3. Characterization

The optical properties of the Liq thin films were investigated by SE in the near-infrared to far ultraviolet spectral region (0.6–6.5 eV). These measurements were conducted using a phase modulated SE tool (Horiba Jobin Yvon, UVISEL, Europe Research Center—Palaiseau, France)using a 20 meV step at an angle of incidence of 70°. Two sets of measurements at different Analyzer Modulator azimuths were necessary for each sample (high accuracy mode) in order to extensively measure the entire range of light polarization changes. The transmittance measurements were performed by the UVISEL ellipsometer at 90° arm setup. The photoluminescence characteristics of the Liq thin films were investigated using the Hamamatsu PL measurement system (C9920-02) (Joko-cho, Higashi-ku, Hamamatsu City, Japan) with an integrating sphere. The surface topography and roughness of the Liq thin films was measured by an Atomic Form Microscope (NT-MDT, NTEGRA, Moscow, Russia).

## 3. Results

### 3.1. Optical Properties

Ellipsometry is an effective method to calculate the dielectric function and to determine the optical properties, as well as the thickness of a large variety of conductive, semiconducting, or dielectric films, through the analysis of the experimentally measured pseudodielectric function <$\tilde{\varepsilon }$(ω)> by applying a suitable theoretical model [18]. The analysis of the measured <$\tilde{\varepsilon }$(ω)> has been performed by the use of a theoretical model that consists of the layer sequence c-Si substrate/SiO_2_ native oxide (2.2 nm)/Liq/air (ambient). For the description of the optical properties of the c-Si and the native oxide SiO_2_, we have used well-established reference values from the literature [18,19]. The quality of the numerical fit was assessed by calculating the root mean square error, which quantifies the deviation between the experimental and theoretical <$\tilde{\varepsilon }$(ω)> values.

The optical properties of Liq have been modeled by the employment of four Tauc–Lorentz (TL) oscillators [20]. The TL model is described by the following expressions:

$$\varepsilon_{2}(\omega )=\frac{AEC(\omega -E_{g}{)}^{2}}{(\omega^{2}-E^{2}{)}^{2}+C\omega^{2}}\frac{1}{\omega },\;\omega >E_{g}$$

(1) $$\varepsilon_{2}(\omega )=0,\;\omega \leq E_{g}$$

(2) $$\varepsilon_{1}(\omega )=\varepsilon_{\infty }+\frac{2}{\pi }P\int_{\omega_{g}}^{\infty }\frac{\xi \varepsilon_{2}(\xi )}{\xi^{2}-\omega^{2}}d\xi$$

In the TL model, the imaginary part $\varepsilon_{2}$(ω) of the dielectric function is determined by multiplying the Tauc joint density of states by $\varepsilon_{2}$(ω) obtained from the Lorentz oscillator model. The real part, $\varepsilon_{1}$(ω), is then derived from the $\varepsilon_{2}$(ω) using Kramers–Kronig integration. This model enables the determination of several key parameters, including the energy position of the fundamental gap (E_g_), electronic transition energy (E), broadening factor (C), and oscillator strength (A). The energy position E corresponds to the Penn gap, whereas the $\varepsilon_{\infty }$ is a constant term that accounts for the existence of electronic transitions above the measured energy region. Although the expressions of $\varepsilon_{1}$(ω) and $\varepsilon_{2}$(ω) in the TL model are empirical, this model can describe and predict the dielectric response and the optical properties of amorphous and crystalline semiconductor films [20,21].

**Figure 1:**
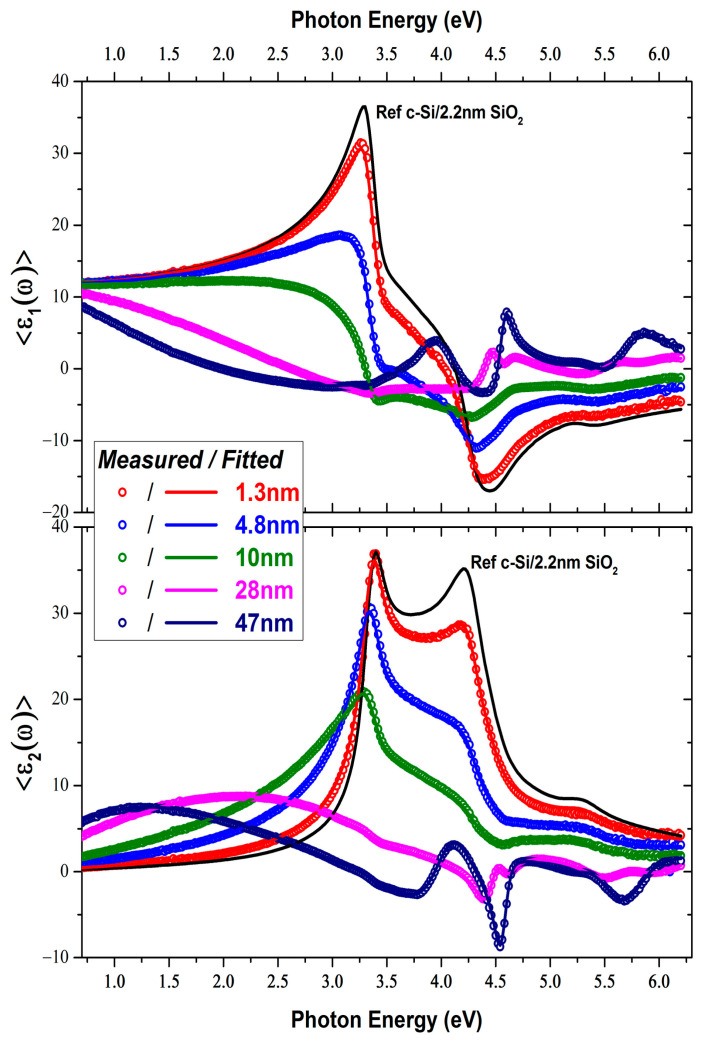
Measured (open circles) and fitted (lines) dielectric function of Liq thin films on c-Si.

Figure 1 shows the real and imaginary parts of the measured <$\tilde{\varepsilon }$(ω)> from the different Liq/c-Si layers together with the fitted curves. Even at the nanoscale, an increase in the thickness of the Liq layer significantly affects the optical response of the Liq/c-Si material system, highlighting the nanometer sensitivity of SE. The optical properties of Liq were extracted from the analysis of the <$\tilde{\varepsilon }$(ω)> acquired from the sample “D” with an estimated thickness of 30 nm according to the QCM. Typically, thin films with thicknesses between 30 and 50 nm maintain the ideal conditions for calculating optical constants via SE, as they provide optimal film morphology and discrete interference effects. In order to take into account the influence of the surface roughness of the deposited Liq samples in their optical response, we implemented the Brugemann Effective Medium Approximation (BEMA) by including in the optical modeling a surface overlayer, which consists of 50% Liq and 50% voids [22]. Nevertheless, the addition of this overlayer did not affect the modeling results, leading to a zero thickness value. This is in agreement with the measured surface roughness by AFM that reported sub-nanometer root mean square (RMS) values, as will be discussed in the next section.

The calculated best-fitted parameters from the <$\tilde{\varepsilon }$(ω)> analysis from sample “C” are shown in Table 2, while the calculated thickness values of the samples, along with the fitting figure of merit (χ^2^), are presented in Table 3. It is important to highlight that ellipsometry modeling assumes a continuous planar layer. Consequently, the calculated thickness results for samples “A” and “B” correspond to the equivalent layer thickness, as will be discussed below.

**Figure 2:**
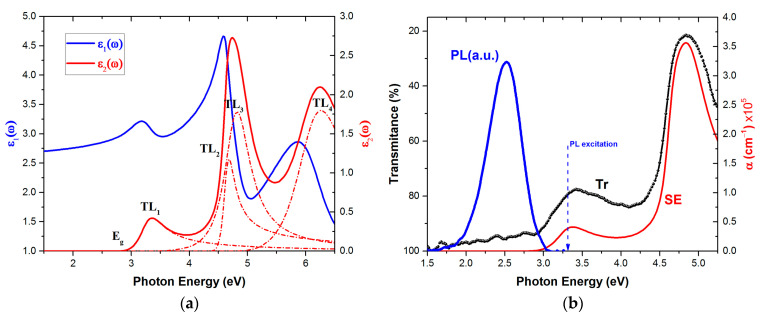
(**a**) Calculated bulk dielectric function of Liq, and (**b**) overlay spectra of Liq PL emission, calculated absorption coefficient, and transmittance (inverted axis to highlight the absorbing properties).

Figure 2a displays the extracted bulk $\tilde{\varepsilon }$(ω) of Liq. The absorption features are located in the UV–fUV spectral region and exhibit three distinct absorption bands. The calculated electronic transitions using the TL model are in good agreement with the DFT calculations of Chiba et al. [23]. The weak absorption peak, which is described by the TL_1_ oscillator parameters at 3.27 eV, is derived from the n–π* transition of the 8-Quinolinol ligand of Liq, which has a partially forbidden nature [23,24]. The strong optical absorptions described by the TL_2,3_ located at 4.65 and 4.79 eV, respectively, can be attributed to the π–π* transition of the ligand. Finally, there is no theoretical study in the literature regarding the TL_4_ electronic transition at 6.13 eV.

It should be noted that the measured <$\tilde{\varepsilon }$(ω)> of the thicker Liq sample has revealed a weak absorption peak at 3.73 eV, consistent with the weak electronic transition reported in the literature for this material [23]. However, incorporating an additional TL oscillator at this energy did not accurately interpret the measured spectrum in that region. Typically, such features are excluded from the fitting procedure, as they cannot define unambiguous oscillator parameters with uncorrelated values between neighboring peaks.

Figure 2b presents the overlayed spectra of transmittance and photoluminescence (PL) measurements in comparison with the calculated absorption coefficient. The transmittance (Tr) and PL measurements were conducted on a sample of the 200 × 200 mm square glass, which housed the mounted Si substrates and was also coated with Liq at the evaporation process. The Tr spectrum of the measured glass/Liq film was divided by the Tr spectrum of a glass substrate reference measurement to isolate the spectral shape of the Liq transmittance characteristics. The measured Tr and SE-calculated absorption coefficient (α) spectra of Liq are in excellent agreement regarding the absorption bands, as well as the E_g_ energy. The tailing of the transmittance spectrum below the E_g_ energy and towards the NIR region is attributed to the gradual variation of the Liq refractive index (Figure 2a), which affects the reflectance of the Liq film in the transparent region. The PL emission of Liq, when excited with energy of 3.32 eV, consists of a single asymmetric peak located in the visible spectral region, with the center of the peak at 2.53 eV.

### 3.2. Film Morphology and Growth

**Figure 3:**
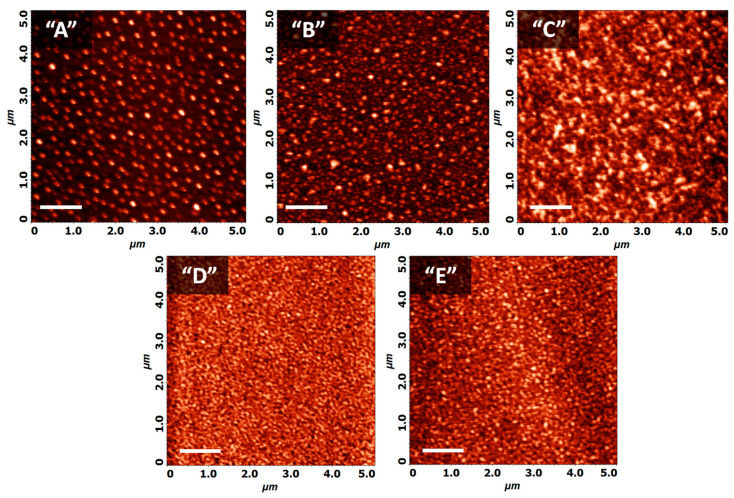
AFM images (5 × 5 μm) of the five (A–E) Liq samples. The white lines indicate a 1 μm scale.

**Figure 4:**
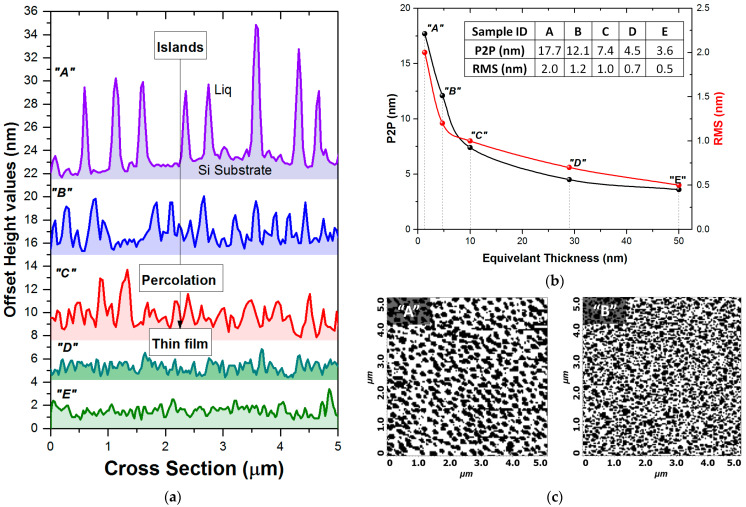
(**a**) AFM cross-sections of the five (A–E) Liq samples, (**b**) P2P and RMS roughness values of the samples (inset: numerical data of RMS and P2P), and (**c**) black-and-white AFM image analysis of samples “A” and “B” for structures with heights greater than 2 nm.

The investigation of the optical properties on thin films is inherently confined to the knowledge of film morphology. Moreover, the selected case studies of samples with progressively increasing film thicknesses can illustrate the growth stages of OVPD Liq films. Figure 3 presents the AFM images of the five (A–E) Liq films on Si samples, while Figure 4a displays the corresponding cross-sectional views of these images and Figure 4b the RMS and peak-to-peak (P2P) analysis.

In the surface nano-topography of sample “A” (with an equivalent layer thickness of 1.3 nm, as determined by SE), we can observe the initial stage of growth, where the Liq material forms islands on the Si substrate. The sample is primarily characterized by platelet-like grains, which are uniformly distributed across the substrate surface (Figure 3 and Figure 4). The size profiles of these grains average 200 nm in diameter and 5–7 nm in height. The uniformity of the Liq grain sizes across the sample indicates a grain size expansion threshold. He et al. recently reported similar Liq structures with a consistent morphology on samples fabricated by gas transport deposition; however, the diameter was around 18 μm [25].

In sample “B”, the Liq surface coverage significantly increases; however, some areas of the substrate remain without deposited material. The grain size does not expand, and the additional deposited material forms new domains on the substrate rather than expanding the islands beyond the 200 nm diameter. Figure 4c presents a black-and-white AFM image analysis of samples “A” and “B”, where the black regions represent material with heights greater than 2 nm, and the white regions correspond to areas with heights less than 2 nm, effectively indicating voids or uncovered substrate. The comparison between samples “A” and “B”, based on image contrast to estimate the area composition, reveals that the coverage of grains taller than 2 nm increases from 44% in sample “A” to 55% in sample “B”. The increased grain density simultaneously reduces the RMS and P2P roughness values (Figure 4b). The RMS roughness of these samples decreases from 2 nm (sample “A”) to 1.2 nm (sample “B”), and the P2P roughness of the grains decreases from 17.7 nm (“A”) to 12.1 nm (“B”).

In the AFM image of sample “C” with an equivalent thickness of 10 nm, the material formed a percolated network of grains. Since there were no samples between 5 and 10 nm, we may assume that the percolation threshold lies within this range, which is also supported by the slope change in the RMS values in Figure 4b. The grain size remains unchanged; however, the RMS and P2P roughness values are further reduced compared to samples “A” and “B”. Although the cross-section indicates the formation of a continuous film, there are large grain boundaries present.

Samples “D” and “E” exhibit similar characteristics, with the material clearly forming a homogeneous and planar thin film. The additional deposited material (compared to sample “C”) has planarized the rough grain boundaries. The differences in the RMS and P2P values fall within the margin of error.

In order to further elaborate on the correlation between the optical properties and structural morphology, the ellipsometric spectra of samples “A”, “B”, and “C” were analyzed using EMA theories. It was assumed that the samples consisted of two-phase composite materials, composed of an unknown volume fraction of voids and Liq, with the composition and thickness of the less dense film numerically calculated. BEMA is commonly used to assess and quantify the homogeneity of deposited films, because it imposes no constraints on the host medium or the phase with the highest percentage, making it suitable for scenarios where the dominant phase may vary depending on the growth stage [26]. However, considering that the morphology of the samples evolves from a separated-grain to a segregated-grain nanostructure during growth, the Maxwell–Garnett EMA was also tested, despite both approximations not fully meeting the criteria for spherical-shaped domains [27]. Interestingly, in both cases, the analysis produced an almost perfect fit, with the best fit indicating a zero percentage of voids. Consequently, the effective dielectric function of the film is identical to that of bulk Liq, and the reduced film density was attributed by the minimization algorithm to the reduced equivalent film thickness. By setting the film thickness of sample “A” to the RMS value of 2 nm, as determined by AFM analysis, the BEMA analysis indicated a 44% volume fraction of Liq material. This result is in remarkable agreement with the AFM coverage analysis of Figure 4c. However, it was not possible to derive an unbiased solution for the simultaneous calculation of both composition and thickness. This limitation may stem from the periodicity and dimensions of the Liq platelet-shaped domains, which produce minimal screening effects on the relative phases of the polarized components of the reflected beam. Consequently, this leads to no observable distortion in the <$\tilde{\varepsilon }$(ω)> spectrum rather than in the optical density. The consistency between the AFM and BEMA results underscores the validity of the structural model, although it highlights the challenges in decoupling the thickness and composition in such analyses [22,28,29].

The proposed optimal layer thickness of Liq films for ETL purposes in OLED devices is 2 nm. According to the presented results, evaporated Liq forms a planar film above the thickness value of 10 nm. However, factors such as the substrate material, evaporation temperature, deposition rate, and other parameters strongly influence the growth and kinetics of thin films, suggesting that achieving a planar 2 nm layer may be feasible. This is a topic for a future work.

## 4. Conclusions

In this work, we reported on the optical properties and growth morphology characteristics of Liq nano-layers on c-Si substrate using the OVPD fabrication process. Five samples of successive Liq film thickness were examined extensively by SE and AFM. The optical properties were modeled according to the TL model and yielded a perfect fit. SE could interpret any thickness variation from 1.3 to 50 nm. AFM analysis reported that, in the early stage of the film’s growth, the material forms islands that are 200 nm wide and 5–7 nm in height. The percolation threshold was found to be within the 5–10 nm range. At 30 nm thickness, the film was observed to be planar and homogeneous, exhibiting sub-nanometer RMS roughness values. The above study of the surface morphology of the Liq EIL and ETL layer is an important parameter to consider when introducing these interfacial layers in OLED devices.

## Figures and Tables

**Table 1:** List of samples studied in this work.

Sample ID	Thickness QCM (nm)
A	2
B	5
C	10
D	30
E	50

**Table 2:** Calculated best-fitted parameters of the electronic transitions of Liq using the Tauc–Lorentz oscillator model.

	TL_j_ Oscillators			
	j = 1	j = 2	j = 3	j = 4
E_g_ (eV)	2.78	4.37	3.31	4.79
A (eV)	8.07	72.81	9.85	36.67
E (eV)	3.27	4.64	4.79	6.13
C (eV)	0.49	0.23	0.54	1.04
$\varepsilon_{\infty }$	1.96			

**Table 3:** Thickness values of the different Liq layers as measured by QCM and as calculated by analysis of the <$\tilde{\varepsilon }$(ω)>. The χ^2^ fitting figure of merit of the analysis is also presented.

	Thickness (nm)				
Measured (QCM)	2	5	10	30	50
Calculated (SE)	1.3	4.8	10	28	48
χ^2^ (SE)	0.36	0.21	0.37	0.26	1.71

## Data Availability

The datasets utilized and/or examined in this study can be obtained from the corresponding author upon making a reasonable request.
